# Clinical Presentation, Anti-microbial Resistance, and Outcome of Salmonella typhimurium Causing Urinary Tract Infections: A Report of Two Cases

**DOI:** 10.7759/cureus.72100

**Published:** 2024-10-22

**Authors:** Aishwarya Ramachandran, Shanthi Mariappan, Uma Sekar

**Affiliations:** 1 Microbiology, Sri Ramachandra Institute of Higher Education and Research, Chennai, IND

**Keywords:** antimicrobial resistance, non-typhoidal salmonella, transient urocolpos, urinary tract infection, vesicovaginal reflux

## Abstract

Non-typhoidal *Salmonella *causing urinary tract infections is uncommon.Their presence in urine is usually suggestive of genitourinary abnormalities, immunosuppression, andother comorbid conditions. The first patient is an 18-year-old female who presented to the Emergency Department with abdominal pain and burning micturition. There were no significant findings in the physical examination Urine culture grew *Salmonella *Typhimuriumwhich was resistant to quinolones and third-generation cephalosporins and positive for the genes encoding their resistance such as bla _OXA-1 _and gyr A respectively. MRI pelvis and CT cystogram revealed findings that were suggestive of vesicovaginal reflux with transient urocolpos. The second patient is a 68-year-old female with a history of CKD and diabetes mellitus who presented for altered sensorium and reduced urine output. Urine culture indicated the presence of *Salmonella *Typhimuriumwhich was susceptible to all the antimicrobial agents tested. This case series highlights that isolation of *Salmonella *Typhimuriumin urine should not be ignored as its presence may indicate the presence of certain predisposing conditions like genitourinary abnormalities, chronic kidney disease and diabetes mellitus. Multidrug resistance is not uncommon among *Salmonella *Typhimurium;* *hence the antimicrobial sensitivity should be assessed and appropriate treatment should be administered.

## Introduction

Non-typhoidal Salmonella (NTS) is a group of entero-invasive organisms that clinically present most commonly as gastroenteritis. Gastrointestinal carrier state, bacteremia, and infections in extraintestinal sites such as meninges, bone, and joints due to NTS have been reported [1,2]. NTS-causing urinary tract infections (UTIs) are uncommon. The estimated frequency of NTS-causing UTIs from various studies ranges from 0.015% to 0.63% [3-5]. The source of NTS infections is contaminated food and the use of inadequate sanitation procedures. NTS causes severe gastroenteritis, bacteremia, meningitis, and septic arthritis. The entry of NTS into the urinary tract is either hematogenous or through urethral duct invasion [6]. Risk factors associated with Salmonella UTIs include underlying urological abnormalities including chronic pyelonephritis, nephrolithiasis, congenital urogenital anomalies such as urethrorectal, or recto-vesical fistula, immunocompromised condition, and chronic illnesses [7]. Here, we present Salmonella Typhimurium isolated in urine samples from two patients with varying clinical findings and antimicrobial susceptibility.

## Case presentation

Case one

An 18-year-old female presented to the emergency room with complaints of non-radiating sharp pricking type of abdominal pain in the left iliac fossa and burning micturition on and off. The patient was obese (weight 100.2 kg, height 158.5 cm, BMI 39.9). Menstrual history was unremarkable and physical examination revealed normal external genitalia. A blood count was performed which showed an elevated total count. Other blood investigations were done and their reports are listed in Table 1. 

**Table 1:** Blood test results performed at the time of admission for case one

Test	Patients’ value	Reference range
Total count	9510	4000-11000 cells/mm^3^
Erythrocytes	4.77	3.8-4.8 mill/cc.mm
Hemoglobin	12.6	12-15 g/dL
Hematocrit	38.5	36-46%
Platelets	3.70	150-400 x 10^3^/μL
Blood urea nitrogen	9	5-18 mg/dl
Creatinine	0.6	0.5-0.9 mg/dL
Sodium	139	136-145 mmol/L
Potassium	4.1	3.5-5.1 mmol/L
Chloride	105	98-107 mmol/L
Bicarbonate	26	22-29 mmol/L

The urine routine revealed normal macroscopic parameters. Urine gram stain showed few pus cells and few gram-negative bacteria. The patient was empirically started on cefoperazone sulbactam 1.5gms intravenously 12th hourly. Semiquantitative urine culture on MacConkey agar had pure growth of 10000 colony-forming units per milliliter (CFU/ml) of non-lactose fermenting colonies. This was identified as Salmonella enterica using matrix-assisted laser desorption-ionization time-of-flight mass spectrometry (MALDI-TOF). Conventional biochemical tests and slide agglutination using specific antisera somatic O, flagella H antigens (Kings Institute, Chennai) helped to identify the isolate as Salmonella *enterica* serovar Typhimurium as specified by the Kauffmann-White scheme.

Antimicrobial susceptibility testing was done using Vitek 2 compact (Biomerieux, France). The isolate was found susceptible to azithromycin, chloramphenicol, cotrimoxazole, imipenem, and meropenem and resistant to ciprofloxacin and ceftriaxone. The isolate was further subjected to PCR with specific primers that are described in Table 2 to characterize the presence of the genes responsible for cephalosporin resistance such as blaCTX-M, bla OXA and bla TEM. Existence of genes responsible for quinolone resistance qnr A,B,C and gyr A,B was also looked for.

**Table 2:** Primers used for the detection of antimicrobial resistance F: Forward primer; R: Reverse primer.

Gene	Primer sequence 5’- 3’	Product size
bla_CTX-M_	^CTX-M-F: CGCTGTTGTTAGGAAGTGTG^ ^CTX -M-R: GGCTGGGTGAAGTAAGTGAC^	_754-bp_
_,_bla_OXA_	_blaOXA F :AAGAAACGCTACTCGCCTGC_ _blaOXA R :CCACTCAACCCATCCTACCC_	_638-bp_
bla_TEM_	_blaTEM__ F:ATGAGTATTCAACATTTCCG_ _blaTEM__R:CTGACAGTT ACCAATGCTTA_	_867-bp_
qnrA	_qnrA- F: ATTTCTCACGCCAGGATTTG_ _qnrA- R: GATCGGCAAAGGTTAGGTCA_	_516-bp_
qnrB	_qnrB- F: GATCGTGAAAGCCAGAAAGG_ _qnrB- R: ACGATGCCTGGTAGTTGTCC_	_619-bp_
qnrC	_qnrS- F: ACGACATTCGTCAACTGCAA_ _qnrS- R : TAAATTGGCACCCTGTAGGC_	_417-bp_
gyrA	_gyrA__- F:__GTTCACCGTCGCATTCT_ _gyrA__- R:__CCCATGACCATCTACAAGC_	_250-bp_

**Figure 1:**
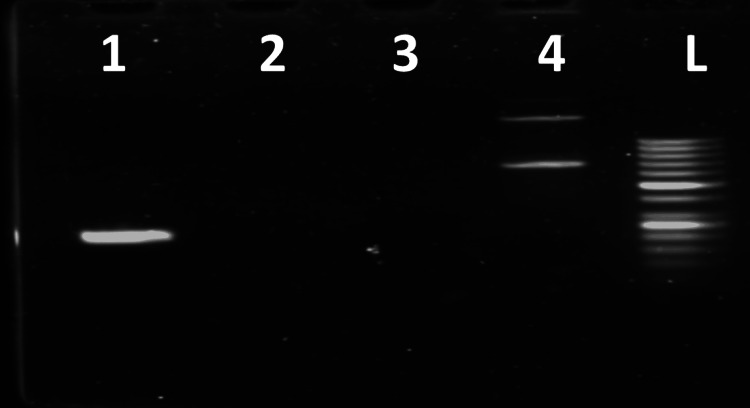
PCR for blaOXA-1 and gyr A Lane 1: gyr A gene ( 250 bp); Lane 2: blaTEM (Negative); Lane 3: blaCTX-M (Negative); Lane 4: blaOXA-1 (638bp); Lane 5: Molecular mass marker (100 bp DNA ladder)

This isolate was positive for bla OXA-1 and gyr A, the genes responsible for cephalosporin and fluoroquinolone resistance respectively (Figure 1).

Further, characterization and sequencing (ABI 3100, Genetic Analyser, Applied Biosystems, USA) of the positive genes were carried out. The Bioedit sequence program was used for analysis and nucleotide sequence similarity searches were performed using the BLAST program. The sequence data generated has been submitted to the NCBI GenBank and an accession number PP941970 has been allotted. After the culture report, the antibiotic was changed to Inj. Meropenem 500 mg every eight hours for seven days. The patient's symptoms related to the urinary tract infection, such as burning micturition and abdominal pain, drastically improved.

**Figure 2:**
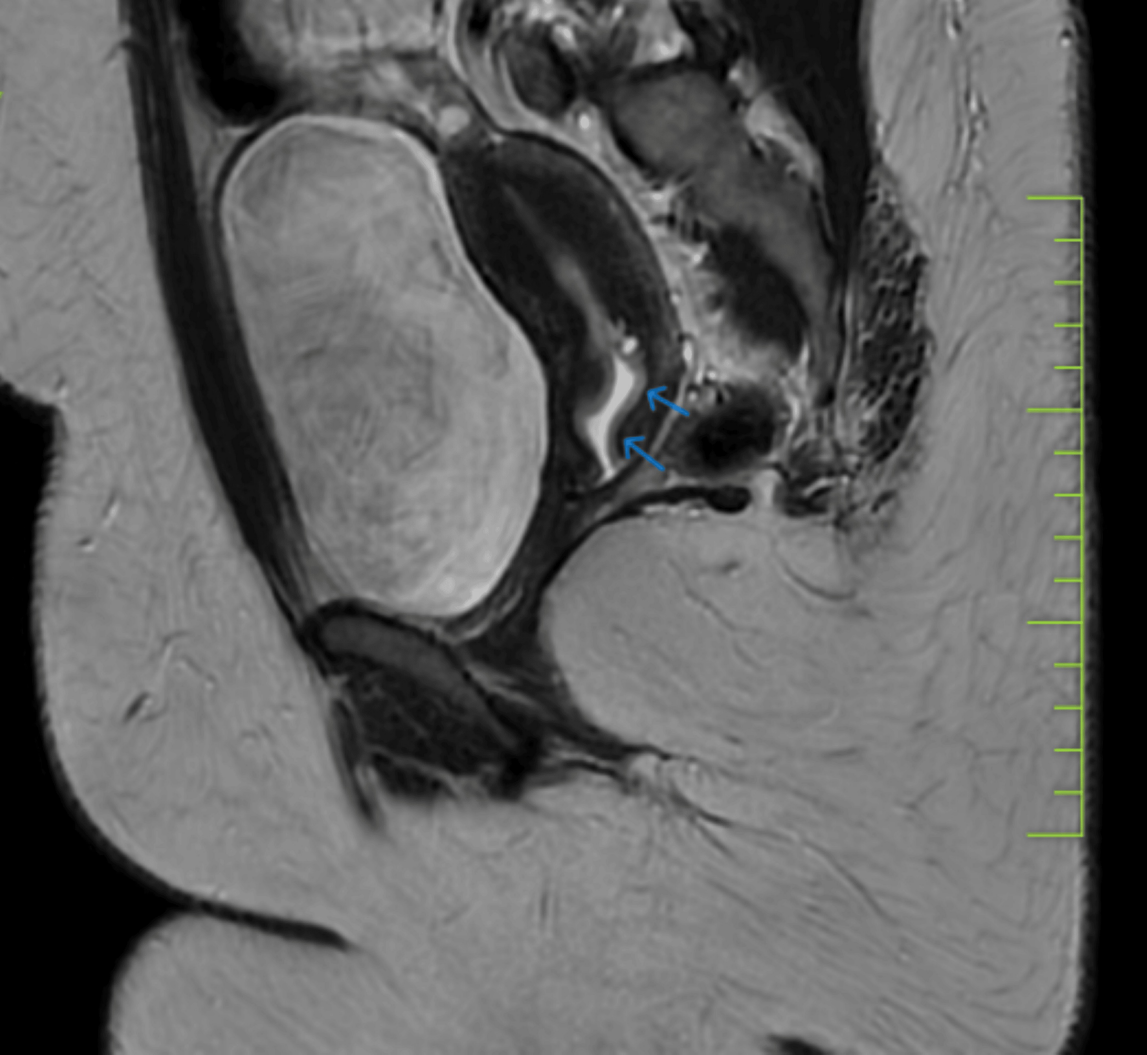
MRI pelvis in full bladder Overdistended bladder with a small amount of fluid within the endocervical canal.

Ultrasound pelvis revealed anechoic collection in the vagina and lower cervical canal which was visible on fully distended bladder but disappeared on voiding. These features raised the possibility of fistulous communication between the genital and urinary tract. MRI pelvis was done on the distended bladder which revealed over distension of the urinary bladder with mild extension of fluid (likely urine) into the endocervical canal (Figure 2).

**Figure 3:**
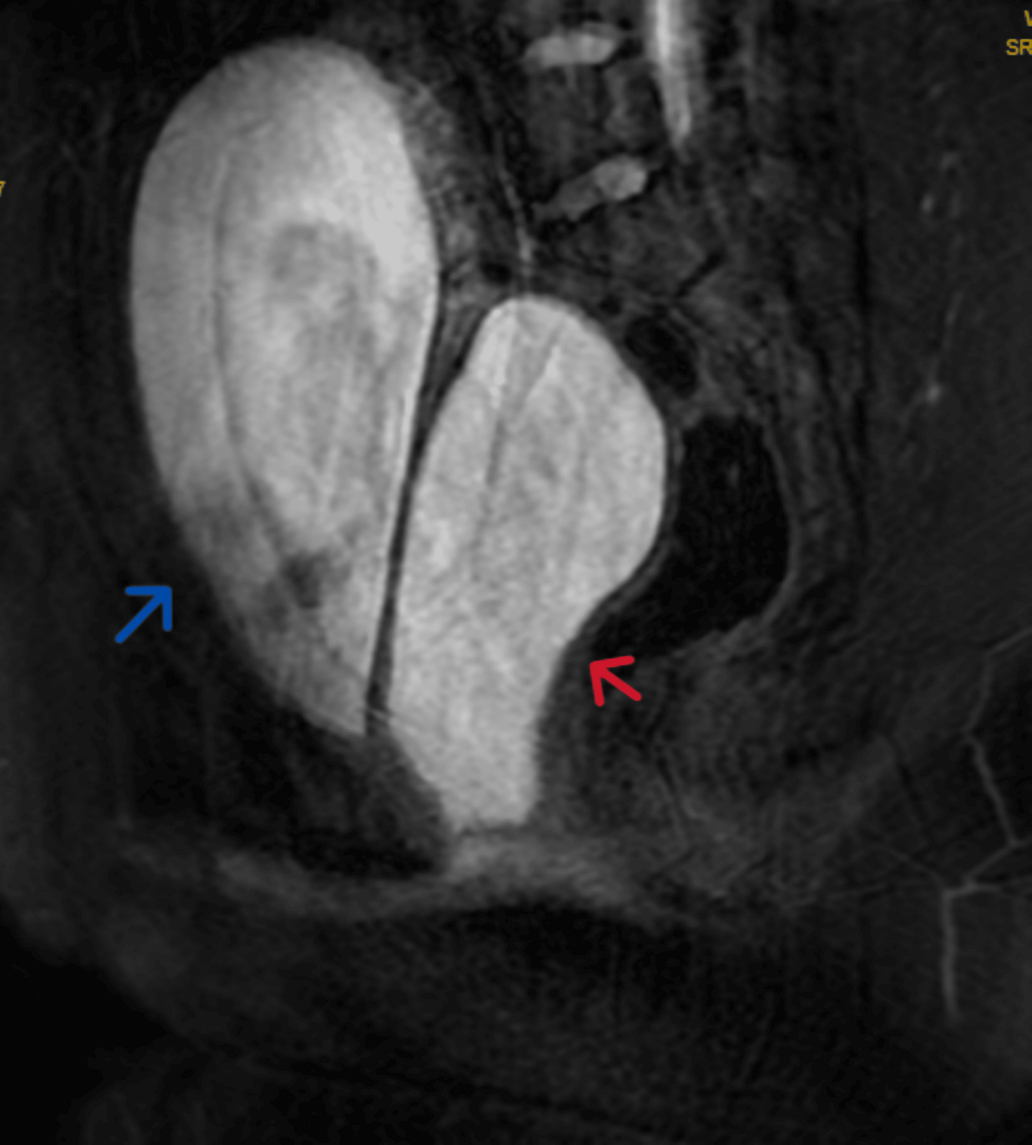
MRI pelvis after partial voiding of urine The blue arrow indicates reduction of the urine volume in the urinary bladder and the red arrow indicates overdistended vagina with urine

Subsequent imaging sequences revealed reduction in the volume of the urinary bladder, with fluid (likely urine) seen within the overdistended vagina (Figure 3).

**Figure 4:**
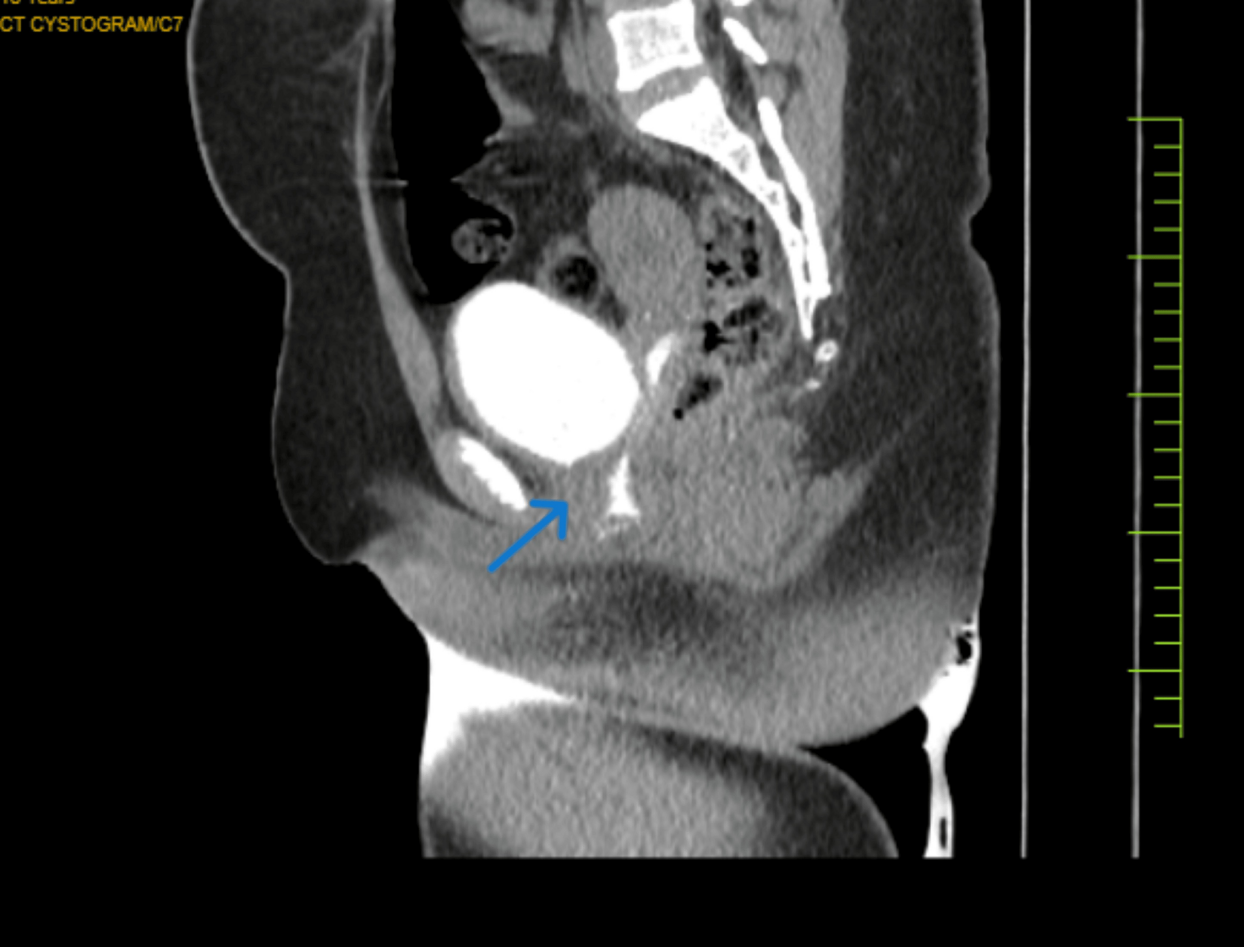
CT cystography Reflux of contrast from urinary bladder into the vagina

In view of these findings, CT cystography was done in which the initial phase of the study revealed an over distended urinary bladder with collapsed vagina (Figure 4).

Subsequent imaging during voiding revealed reflux of contrast from urinary bladder into the vagina. These imaging findings were suggestive of vesicovaginal reflux (VVR) with transient urocolpos.

Case two

A 68-year-old female with comorbid conditions such as chronic kidney disease (CKD) and diabetes mellitus presented with complaints of drowsiness and reduced urine output. On examination, the patient was disoriented and responding only to painful stimuli. The patient had been managed medically for CKD and diabetes mellitus for eight years. All routine investigations were done and are presented in Table 3. Potassium, urea, and creatinine levels were elevated at 6.6 mmol/L 68 mg/dl and 5.2 mg/dl, respectively. Low hemoglobin and low hematocrit 8.2 g/dl and 23%, respectively, were observed in this patient.

**Table 3:** Blood test results performed at the time admission for case two

Test	Patients’ value	Reference range
Total count	8770	4000-11000 cells/mm^3^
Erythrocytes	4.77	3.8-4.8 mill/cc.mm
Hemoglobin	8.2	12-15 g/dL
Hematocrit	23	36-46%
Platelets	2.53	150-400 x 10^3^/μL
Blood urea nitrogen	68	5-18 mg/dl
Creatinine	5.2	0.5-0.9 mg/dL
Sodium	126	136-145 mmol/L
Potassium	6.6	3.5-5.1 mmol/L
Chloride	96	98-107 mmol/L
Bicarbonate	15	22-29 mmol/L

**Figure 5:**
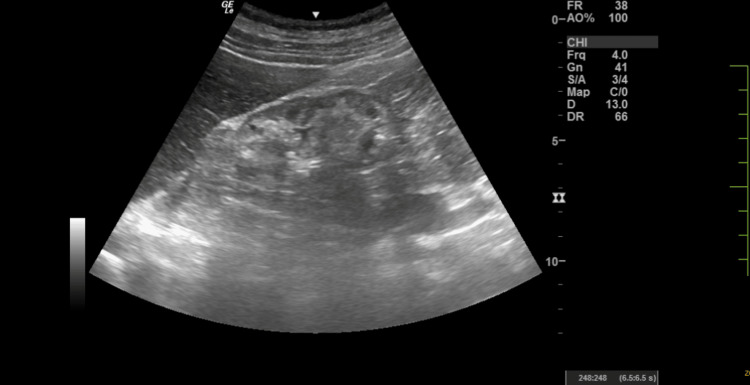
Ultrasound abdomen showing the right kidney The right kidney appears small in size and corticomedullary differentiation poorly maintained

**Figure 6:**
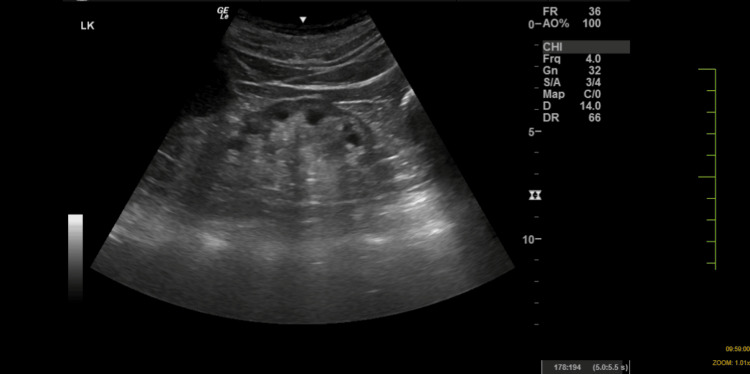
Ultrasound abdomen showing the left kidney The left kidney appears small in size with poor differentiation of the corticomedullary junction.

Ultrasound abdomen showed bilateral kidneys shrunken with poor corticomedullary dysfunction (Figures 5, 6).

Urine gram stain had few pus cells and little gram-negative bacteria. Urine semiquantitative culture grew 10000 colony-forming units per milliliter (CFU/ml) of non-lactose fermenting colonies on Mac conkey agar. This isolate was also identified as Salmonella enterica serotype typhimurium using the same automated and conventional identification methods that are mentioned above. Antimicrobial susceptibility testing was done using Vitek 2 compact (Biomerieux, France). The isolate was found susceptible to chloramphenicol, azithromycin, cotrimoxazole, ciprofloxacin, third-generation cephalosporins and carbapenems. The patient was managed medically and started on tablet cefixime 200 mg BD. Patient’s sensorium gradually improved, creatinine levels showed a reducing trend and urine output increased.

## Discussion

NTS species majorly causes gastroenteritis which may be accompanied or followed by complications such as bacteremia, focal infections and other invasive diseases Salmonellae causing urinary tract infections is either due to direct urethral invasion associated with genitourinary abnormalities or immunosuppressed conditions such as HIV, patients on steroids and chronic conditions such as diabetes mellitus, neoplasms of the kidney, lupus nephritis and chronic hemodialysis [8,9]. NTS in urine is rare and it is considered as a surrogate marker of an underlying anatomical abnormality of the genitourinary tract [10].

Reflux of urine into the vaginal vault during micturition occurring in both supine and upright positions is referred to as VVR. Urocolpos refers to distension of the vagina due to accumulation of urine resulting commonly from vesicovaginal fistula or reflux. Urogenital malformations like VVR and obstructive vaginal lesions are commonly associated with urocolpos. Other risk factors include pelvic floor dysfunction, obesity, and improper toilet training. The clinical presentation of VVR is diverse, including abdominal pain, abdominal distension, vulvovaginitis, daytime enuresis, and recurrent urinary tract infections. Common radiological diagnostic modalities used for VVR are Ultrasound, pelvic MRI, and voiding cystourethrography [11]. NTS has also been isolated in patients with chronic conditions such as diabetes mellitus.

NTS causing urinary infection warrants early institution of antibiotics. Numerous reports of multidrug-resistant (MDR) NTS causing severe febrile illness in Sub-Saharan Africa have emerged. Antimicrobial resistance among NTS in animal and human blood isolates ranges from 30-70% [12,13]. However, there are not many MDR NTS reported in urine samples from Asia, particularly India [14].

In routine clinical practice, third-generation cephalosporins and fluoroquinolones are preferred in the treatment of salmonella UTIs owing to their excellent tissue penetration, high intracellular concentrations, increased bactericidal activity, and urinary excretion as an active drug. Among Salmonellae-causing UTIs, Salmonella Typhimurium has been the most frequently reported serotype [15]. Salmonella Typhimurium isolated from the first patient co-harbored ESBL genes on mobile genetic elements and antimicrobial resistance genes for fluoroquinolones. This decreases the available options for antimicrobial therapy as this isolate possesses a plasmid containing multiple antimicrobial resistance genes.

In a study conducted in Spain, NTS in urine was detected in 19 patients. Among these patients, eight (42.1%) patients had diabetes mellitus and eight patients (42.1%) had urologic abnormalities [16].

Thus, from this study, it is understood that isolation of NTS in urine emphasizes the need for a detailed genito-urinary system examination and assessment of comorbid conditions. In cases of UTIs associated with urinary tract anomalies and chronic conditions, it is prudent to choose the appropriate empirical antimicrobial agent while waiting for the microbiological culture report to eliminate the infection and reduce the length of the hospital stay. 

## Conclusions

This report highlights the importance of screening for genitourinary anomalies and suspicion of underlying chronic diseases or immunosuppression in the presence of NTS in urine. Further, isolation of rare isolates such as NTS in urine should not be ignored and their resistance to drugs with high urinary concentration is not uncommon. This study enforces the importance of choosing appropriate antibiotics after in vitro sensitivity data in such conditions as there are no specific antimicrobial guidelines for NTS in urine. This would help prevent complications such as potential bacteremia and invasive salmonella infections and recurrences.
